# Description of *Eucyclops tziscao* sp. n., *E. angeli* sp. n., and a new record of *E. festivus* Lindberg, 1955 (Cyclopoida, Cyclopidae, Eucyclopinae) in Chiapas, Mexico

**DOI:** 10.3897/zookeys.351.5413

**Published:** 2013-11-15

**Authors:** Martha Angélica Gutiérrez-Aguirre, Nancy Fabiola Mercado-Salas, Adrián Cervantes-Martínez

**Affiliations:** 1 Universidad de Quintana Roo (UQROO), Unidad Cozumel, Av. Andrés Quintana Roo s/n, 77600, Cozumel, Quintana Roo México; 2 El Colegio de la Frontera Sur (ECOSUR). Unidad Chetumal. Av. Centenario Km. 5.5. Chetumal, Quintana Roo 77014. México

**Keywords:** Copepoda, description, freshwater, free-living, Neotropical

## Abstract

Two new species of the freshwater cyclopoid genera *Eucyclops* are described, *Eucyclops tziscao* sp. n. and *E. angeli* sp. n. Both species belong to the *serrulatus*-group defined by morphological features such as: the presence of distal spinules or hair-like setae (groups N1 and N2) on frontal surface of antennal basis; the fourth leg coxa with a strong inner spine that bears dense setules on inner side, yet proximally naked (large gap) on outer side; and a 12-segmented antennule with smooth hyaline membrane on the three distalmost segments. *Eucyclops tziscao* **sp. n.** is morphologically similar to *E. bondi* and *E. conrowae* but differs from these species in having a unique combination of characters, including a caudal ramus 4.05±0.25 times as long as wide, lateral seta of Enp3P4 modified as a strong, sclerotized blunt seta, coxal spine of fourth leg with inner spinule-like setules distally, and sixth leg of males bearing a strong and long inner spine 2.3 times longer than median seta. *Eucyclops angeli* **sp. n.** can be distinguished by an unique combination of morphological features: the short caudal ramus; the long spine on the sixth antennular segment of A1; the presence of one additional group of spinules (N12’) on the caudal surface of A2; the presence of long setae in females, or short spinules in males on the lateral margin of fourth prosomite; the strong ornamentation of the intercoxal sclerite of P4, specially group I modified as long denticles; the distal modified setae of Exp3P3 and Exp3P4 in females and males; and the short lateral seta of P5. Finally, we report on a new record of *E. festivus* in México, and add data on morphology of the species.

## Introduction

*Eucyclops* Claus, 1893 is the largest genera of the subfamily Eucyclopinae, currently comprising up to 108 species and subspecies distributed mainly in the tropics (Dussart and Defaye 2006, Alekseev and Defaye 2011). Because of its high diversity, this group is one of the taxonomically most challenging genera within the freshwater Copepoda, with several problematic taxa and with high intraspecific variation in some species groups. Also, many *Eucyclops* species are poorly described, therefore the taxonomic position of them remain uncertain (Collado et al. 1984, Reid 1985, Ishida 1997, Suárez-Morales 2004, Mercado-Salas et al. 2012). Nonetheless significant attempts have been made to revise the most problematic species groups in the genus: Ishida (1997, 2001, 2002, 2003) revised the “*serrulatus*-like” and “*speratus*-like species” from Japan; while Alekseev et al. (2006) and Alekseev and Defaye (2011) provided a world-scale overview of the taxonomy and zoogeography of the *Eucyclops serrulatus*-group. These studies revealed the diagnostic significance of many previously neglected characters [e.g. ornamentation of the antennal basis and swimming legs (the fourth leg in particular), or pore signature] which might be also useful in the delineation of other taxa.

In the Americas there are more than 800 records of the genus, corresponding to 28 nominal species, most of which are distributed in the eastern of United States, México, Argentina, and Brazil. Approximately 38% of these records have been assigned to the problematic taxa *Eucyclops serrulatus* (Fischer, 1851) and *Eucyclops agilis* (Koch, 1838) (Lindberg 1955, Collado et al. 1984, Reid 1985, Suárez-Morales 2004, Bruno et al. 2005, Frisch and Threlkeld 2005, Gaviria and Aranguren 2007, Elías-Gutiérrez et al. 2008, Suárez-Morales and Walsh 2009, De los Ríos et al. 2010).

In México 13 species have been recorded so far: *Eucyclops agilis* (synonym of *Eucyclops serrulatus*), *Eucyclops bondi* Kiefer, 1934; *Eucyclops chihuahuensis* Suárez-Morales & Walsh, 2009; *Eucyclops conrowae* Reid, 1992; *Eucyclops cuatrocienegas* Suárez-Morales & Walsh, 2009; *Eucyclops elegans* (Herrick, 1884), *Eucyclops festivus* Lindberg, 1955; *Eucyclops leptacanthus* Kiefer, 1956; *Eucyclops pectinifer* (Cragin, 1883), *Eucyclops prionophorus* Kiefer, 1931; *Eucyclops pseudoensifer* Dussart, 1984; *Eucyclops serrulatus* (probably *Eucyclops pectinifer*) and *Eucyclops torresphilipi* Suárez-Morales, 2004 (Lindberg 1955, Zamudio-Valdéz 1991, Suárez-Morales and Reid 1998, Suárez-Morales 2004, Mercado-Salas 2009).

Grimaldo-Ortega et al. (1998), Elías-Gutiérrez (2000), Rodríguez-Almaraz (2000), Suárez-Morales (2004), and Mercado-Salas (2009) have documented morphological differences between the Mexican populations and the original descriptions of those *Eucyclops* taxa, which indicated that a few undescribed species might have been hidden under the name of the “cosmopolitan” species. Also Suárez-Morales (2004) and Suárez-Morales and Walsh (2009) mentioned that the species richness of *Eucyclops* in Mexico could be underestimated.

In agreement with this assumption, we describe two new *Eucyclops* species and report the new record of a third one in Chiapas, México. Chiapas is one of the hydrologically richest regions in Mexico, with numerous and diverse aquatic environments such as rivers, lakes, lagoons, reservoirs and a large coastline (Velázquez-Velázquez 2011). Although in recent years substantial progress has been made in the knowledge about the freshwater fauna in this region (mainly fishes), hardly anything is known about other animal groups, such as the crustaceans for instance (Velázquez-Velázquez 2011).

The knowledge of the copepod fauna in Chiapas and the cyclopoids in particular, is almost null; only eighteen species have been recorded (Suárez-Morales 2004, Gutiérrez-Aguirre et al. 2006, Elías-Gutiérrez et al. 2008, Gutiérrez-Aguirre and Cervantes-Martínez 2013). Thus the goal of this study is to contribute to the basic knowledge of the freshwater Copepoda of this region.

## Methods

**Figure 1.**
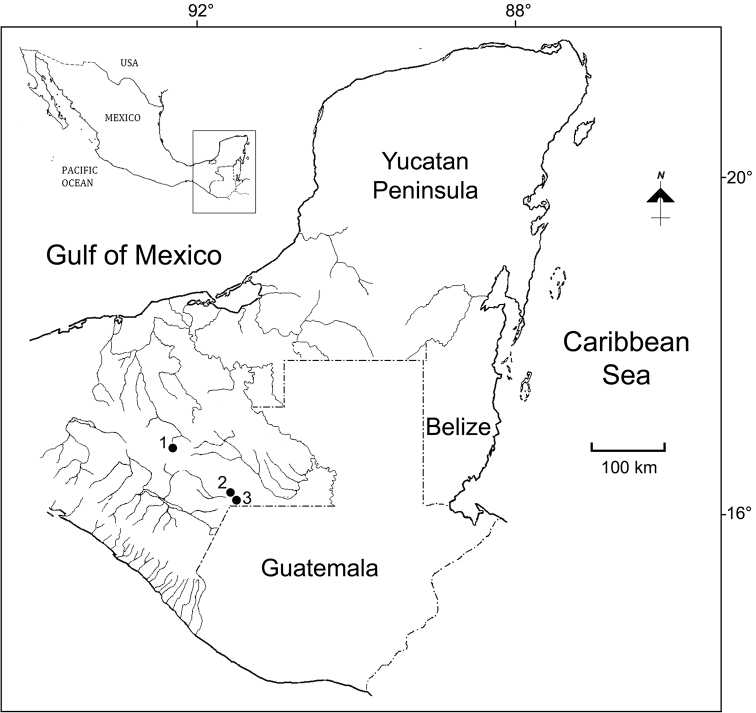
Collecting sites at Chiapas, México. **1** San Cristóbal de las Casas **2** Pond 3 to Laguna Montebello **3** Laguna Tziscao.

The samples were collected from the limnetic zone of Laguna Tziscao, as well as from the littoral of some ephemeral or permanent reservoirs in Chiapas (México) in 2000-2001. The collecting sites (1500 masl) are shown in Fig. 1. The samples were collected by standard plankton net of 0.05 mm mesh-size, performing near-shore and limnetic plankton trawls. The biological specimens were fixed and preserved in 70% ethanol, and then processed for identification following the techniques described by Reid (2003). All adult *Eucyclops* in the samples were identified to species level.

The specimens were dissected with tungsten needles and the appendages were mounted in glycerin for taxonomic analysis. The mouth parts, swimming legs, and other taxonomically important structures were illustrated with the aid of a camera lucida. Specimens were deposited in the Collection of Zooplankton of ECOSUR at Chetumal, Mexico (ECO-CH-Z), in the Collection of Copepoda of the Muséum National d’Histoire Naturelle (MNHN-IU) Paris, and in the Colección Nacional de Crustáceos (CNCR) del Instituto de Biología, Universidad Nacional Autónoma de México. Examination of the specimens has been performed following the current methods used in the morphological investigations of Eucyclopinae (Alekseev 2000, Alekseev et al. 2006).

Abbreviations used in the descriptions are as follows: A1, antennule; A2, antenna; P1-P4, first to fourth swimming legs; P5, fifth leg; Exp, exopod; Enp, endopod; s, seta(e); ae, aesthetasc; sp, spine; Bsp, basis; Fu, caudal ramus. The terminology used for the armament of the antenna and swimming limbs is what was proposed by Alekseev et al. (2006) and Alekseev and Defaye (2011).

## Results

### Order Cyclopoida Burmeister, 1834
Family Cyclopidae Dana, 1846
Subfamily Eucyclopinae Kiefer, 1927
Genus *Eucyclops* Claus, 1893

#### Eucyclops tziscao

Mercado-Salas sp. n.

http://zoobank.org/967E9152-65BF-4F59-9D91-ACFF91C7834B

http://species-id.net/wiki/Eucyclops_tziscao

Figs 2 –4

##### Synonym.

*Eucyclops bondi*: Gutiérrez-Aguirre and Cervantes-Martínez (2013), Table 1.

**Table 1.** Comparative material: locality and data on slide labels.

Species	Slide reference number
*Eucyclops bondi*	SMNK 02079, female sp. n., Trou Caiman, Haiti. 16.02.1933
	SMNK 02080, male, Typus, Trou Caiman, Haiti, 16.02.1933
	SMNK 02393, female, Laguna Rincon, Haiti
	SMNK 02394, female, Laguna Rincon, Haiti
*Eucyclops conrowae*	USNM-251325, holotype, Id: Janet W. Reid; Collector: R. Conrow; Shark River
	Slough, Everglades National Park, Florida, United States. 1986.
	USNM-251327, paratype; Id: Janet W. Reid; Collector: R. Conrow; Shark River
	Slough, Everglades National Park, Florida, United States. 1986.

##### Material examined.

Holotype: Adult *♀* specimen dissected, mounted in glycerin sealed with Entellan (ECO-CH-Z-08970). Allotype: Adult *♂*, dissected, mounted in glycerin sealed with Entellan (ECO-CH-Z-08971). Paratypes: Eight adult *♀♀*, one adult *♂* and two copepodites undissected ethanol-preserved (90%) (ECO-CH-Z-08972); three adult *♀♀*, undissected, ethanol-preserved (90%) (CNCR-27840). The types were collected at 15.April.2000 by A. Cervantes-Martínez, M. A. Gutiérrez-Aguirre and M. Elías-Gutiérrez.

##### Comparative material.

To complement the morphological analysis, we also examined the type specimens of *Eucyclops bondi* deposited in the Staatliches Museum für Naturkunde Karlsruhe (SMNK) from Kiefer’s collection, and the type specimens of *Eucyclops conrowae* deposited in the National Museum of Natural History Smithsonian Institution, in Washington D. C. (USNM) (Table 1).

##### Type locality.

Laguna Tziscao, Chiapas, México (16°05'19"N, 91°40'10"W). At sampling the maximum depth was 74.5 m, the water temperature 22°C, and the dissolved oxygen 6.6 mg L^-1^. The system is considered as one of the deepest, oligotrophic lagoons in the southern Mexico, with karstic origin, located in Lagunas de Montebello National Park which belongs to the Usumacinta biogeographic province.

##### Etymology.

The species name is a noun in apposition that makes reference to the Lagoon where the species was collected from. Tziscao (*Tz’isk’a’aw*) is a term composed by two words in the chuj local language (one of the Mayan languages), and it refers to the stone bridge made by hand by the first settlers of the community.

##### Description.

**Figure 2.**
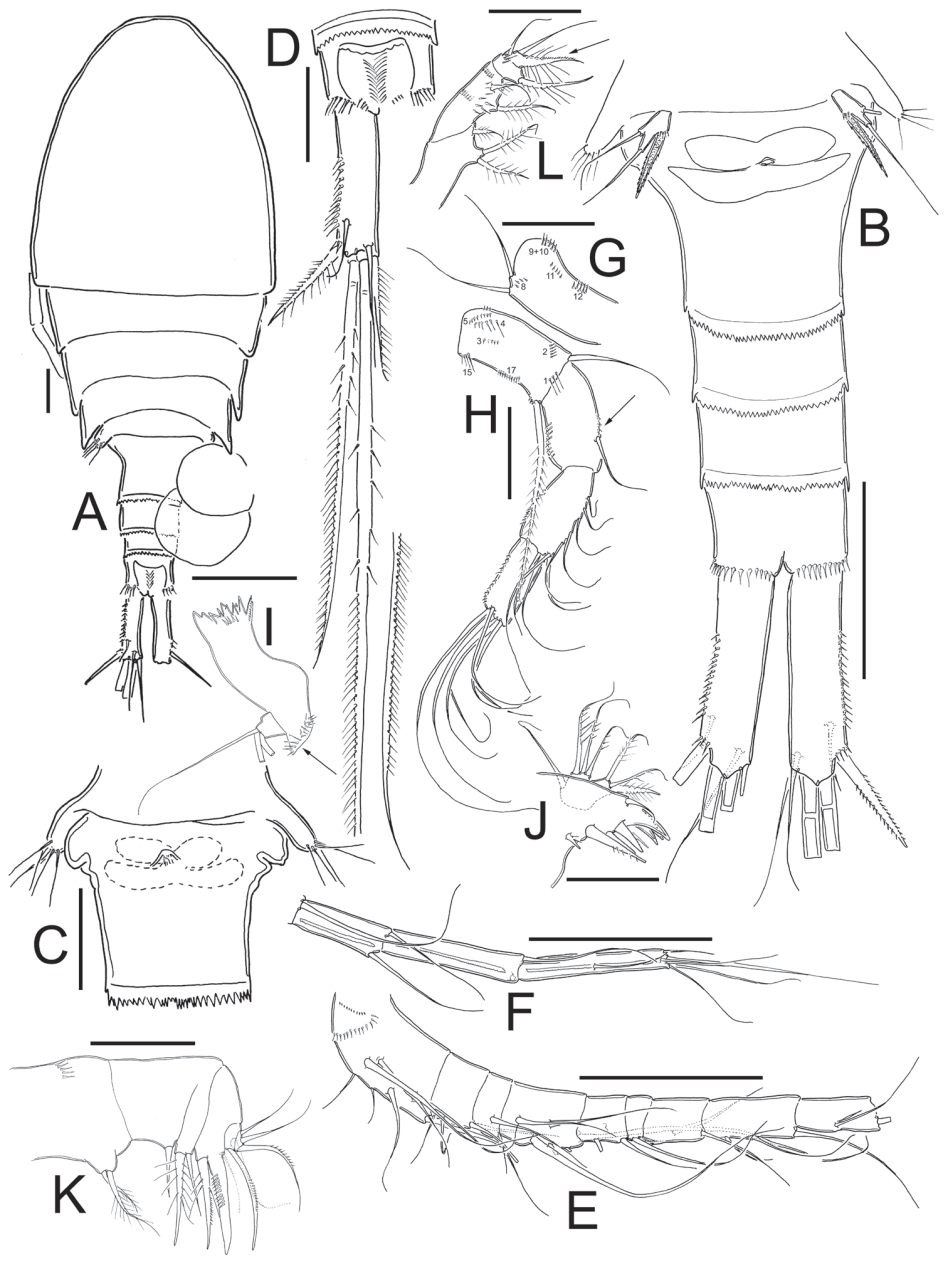
*Eucyclops tziscao* sp. n. **A, C, D** paratype **B, E–L** holotype from Laguna Tziscao, Chiapas. **A** Habitus, dorsal **B** Urosome **C** Genital double-somite, ventral **D** Anal somite and caudal ramus, dorsal **E** Antennule, segments 1–9 **F** Antennule, segments 10–12 **G** Antenna, caudal **H** Antenna, frontal **I** Mandible **J** Maxillule, caudal **K** Maxilla, frontal **L** Maxilliped, frontal. Scales bars: **K** = 20 µm; **A, C, D, G, H, I, J, L** = 50 µm; **B, E, F** = 100 µm.

*Female*: Habitus as in Fig. 2A. 620 µm of total body length excluding caudal setae. Prosome expanded at first and second somite, representing 61% of total body length symmetrical in dorsal view. Five-segmented urosome relatively elongated, urosomal fringes strongly serrated (Fig. 2B); posterior margin of anal somite with one row of long spinules. Genital double-somite (Fig. 2C) symmetrical, carrying paired egg sacs. Lateral arms of seminal receptacle rounded on posterior margin. Genital double-somite 1.3 times as long as wide. Anal somite with hair-setae in anal opening, anal operculum serrated (Fig. 2D). Caudal ramus 4.0 times as long as width; inner margin naked, strong spines on the lateral margin (serra) extending 40% of ramus length (Fig. 2D). Dorsal seta (VII) short: 0.65 times the length of caudal ramus, and 1.1 times as long as outermost caudal seta (III). Ratio of innermost caudal seta (VI)/outermost caudal seta (III) is 1.2. Lateral caudal seta (II) inserted at 71% of caudal ramus. All the terminal caudal setae plumose.

*Antennule* (Figs 2E, F): 12-segmented, reaching from middle to distal margin of third prosomite; last three segments with finely denticulate hyaline membrane at distal margin. Armament per segment as follows (s= seta, ae= aesthetasc, sp= spine):1(8s), 2(4s), 3(2s), 4(6s), 5(4s), 6 (1s+1sp), 7(2s), 8(3s), 9(2s+1ae), 10(2s), 11(3s), 12(8s). Two rows of spines on first segment, first row with small spinules and second row with stronger and longer spinules. Spine on sixth segment reaching middle of seventh antennular segment.

*Antenna* (Figs 2G, H): Coxa (no seta), basis (2s + 1 seta representing Exp), plus 3-segmented Enp (first to third Enp with 1, 9 and 7 setae, respectively). Basis ornamented with: N1 (3-4 hair-setae), N2 (5 small spinules), N3, N4, N5, N15, and N17 on frontal surface (Fig. 2H); and N8, N9+10, N11, and N12 on caudal surface (Fig. 2G). First to third endopodites with dense rows of spinules along lateral margins; Enp1 with an additional row of 5 spinules along medial margin below seta (arrowed in Fig. 2H).

*Labrum*: Distal margin toothed.

*Mandible* (Fig. 2I): With seven teeth on gnathobase. Innermost margin with one spinulose seta. Row of 6 spinules in middle, below gnatobase. Palp with two long and one short seta, group of spinules near to palp (arrowed in Fig. 2I).

*Maxillule* (Fig. 2J): Precoxal arthrite with naked surface, with three strong chitinized distal claws and one spiniform seta on caudal side. Palp unarmed, Enp with three setae (two smooth setae subequal in size, and one plumose shorter seta), Exp with three setae and Bsp with one plumose seta.

*Maxilla* (Fig. 2K): Praecoxa and coxa partially fused. Praecoxa with endite bearing two setae and a transverse row of small spinules on frontal surface. Coxa naked, bearing one biserially plumose seta. Distal coxal endite well developed, with two apical setae, one strong and furnished with spinules and other one noticeably thicker and longer. Claw-like basal endite with one row of spinules on inner margin, one chitinized armed seta inserted in front of basal “claw” and one seta inserted at base of claw-like endite on caudal surface. Endopod with a single segment bearing five setae.

*Maxilliped* (Fig. 2L): Syncoxa with three setae. Basis with two sub equal setae, plus 8 long spinules on frontal surface. Two transverse rows of small spinules, each with 6-8 elements arranged in semi-circular pattern on caudal surface. Endopod two-segmented: Enp1 with one long seta and one transverse row of 5 spinules on frontal surface. Enp2 with three setae, the longest fused to Enp2 and biserially plumose on the proximal half, the distal half ornamented with small spinules in caudal surface (arrowed in Fig. 2L).

*Legs 1–4*: Endopods and exopods of all swimming legs three-segmented. Armature formula of swimming legs as in Table 2.

**Table 2.** *Eucyclops tziscao* sp. n. Setation formula of the swimming legs in female, and male (spine in Roman numerals, seta in Arabic numerals).

	Coxa	Basis	Exp	Enp
P1	0-1	1-I	I-1; I-1; III-5	0-1; 0-2; 1-I-4
P2	0-1	1-0	I-1: I-1; IV-5	0-1; 0-2; 1-I-4
P3	0-1	1-0	I-1; I-1; IV-5	0-1; 0-2; 1-I-4
P4	0-1	1-0	I-1; I-1; III-5	0-1; 0-2; 1-II-2

**Figure 3.**
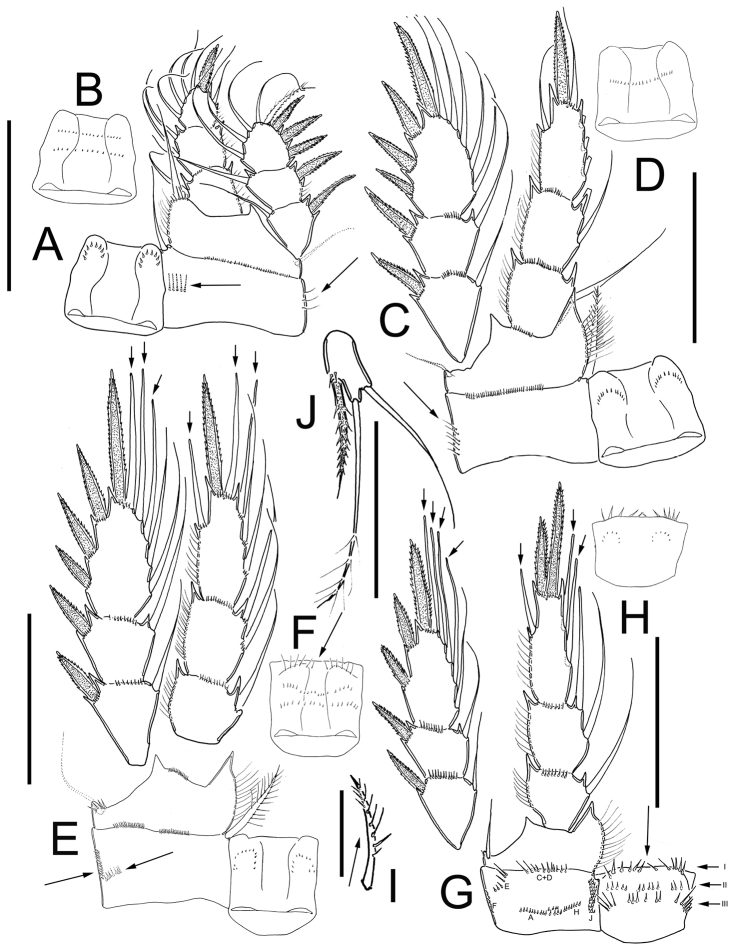
*Eucyclops tziscao* sp. n. Holotype from Laguna Tziscao, Chiapas. **A** P1, frontal **B** Intercoxal sclerite of P1, caudal **C** P2, frontal **D** Intercoxal sclerite of P2, caudal **E** P3, frontal, Exp and Enp separated **F** Intercoxal sclerite of P3, caudal **G** P4, caudal **H** Intercoxal sclerite of P4, frontal **I** Coxal spine P4 **J** P5. Scales bars: **I**= 25µm, **J**= 50 µm; **A–H** = 100 µm.

*Leg 1* (Figs 3A, B): Intercoxal sclerite with one row of spinules arranged in a semi-circle on each side of frontal surface (Fig. 3A); caudal surface with two transversal rows of tiny spinules, distal margin with two rounded chitinized projections (Fig. 3B). Coxa with strong biserially plumose inner coxal seta. Coxa with one row of hair-setae on outer margin and one transverse row of hair-setae next medial margin (arrowed in Fig. 3A). Inner basal seta reaching middle of Enp3, 0.76 times as long as Enp.

*Leg 2* (Fig. 3C, D): Intercoxal sclerite with two groups of small spinules arranged in semi-circle on each side of frontal surface (Fig. 3C), and one transverse row of spinules in middle on caudal surface (Fig. 3D). Distal margin of intercoxal sclerite with two rounded chitinized projections. Coxa with strong biserially plumose inner coxal seta. Coxa with one row of hair-setae along outer margin on frontal surface (arrowed in Fig. 3C) small spines next insertion of Enp.

*Leg 3* (Fig. 3E, F): Intercoxal sclerite with two groups of small spinules on frontal surface (Fig. 3E) caudal surface of intercoxal sclerite with three rows of spinules: distal row bearing long hair-like spinules at each side (arrowed in Fig. 3F), middle and proximal rows with tiny spinules. Distal margin with two slightly rounded projections. Coxa bearing strong biserially plumose inner coxal seta, frontal surface with one row of tiny spinules along outer (lateral) margin, and one transverse row of spinules on caudal surface (arrowed in Fig. 3E). Modified setae on Enp3 and Exp3 (arrowed in Fig. 3E). Tiny spinules at insertion of all setae of Enp and all spines of Exp.

*Leg 4* (Figs 3G–I): Intercoxal sclerite with rows I, II, and III on caudal surface. Row I with strong spinules on each side and a small gap. Row II with small spinules divided into three sections with small gaps between them. Row III divided into three sections, the first section with 5 long spinules, the middle section with 6 small strong spinules, and the third section with 5 long spinules (Fig. 3G). Frontal surface of intercoxal sclerite with two groups of tiny spinules arranged in semi-circle on each side (Fig. 3H). Caudal surface of coxa with spinules groups A-C, and E-F-H-J. Inner coxal spine (seta) with heteronomous setulation: proximally with long hair-like setules, distally with spinule-like setules; outer edge of coxal spine with three spinule-like setules distally, naked proximally (arrowed in Fig. 3I). Enp3P4 3.0 times as long as wide; inner spine 1.4 times as long as outer spine and 1.1 times as long as segment; outer spine 0.70 times as long as segment. Lateral seta of Enp3P4 inserted at 66% of the total length of segment. Modified setae on Enp3 and Exp3 (arrowed in Fig. 3G). All setae of exopod with tiny spinules at insertion.

*Leg 5* (Fig. 3J): One free segment subrectangular, 2.1 times longer than wide; bearing one inner spine and two setae; median seta about 1.3 times longer than outer seta and 1.8 times longer than inner spine. Inner spine 1.7 times as long as segment.

**Figure 4.**
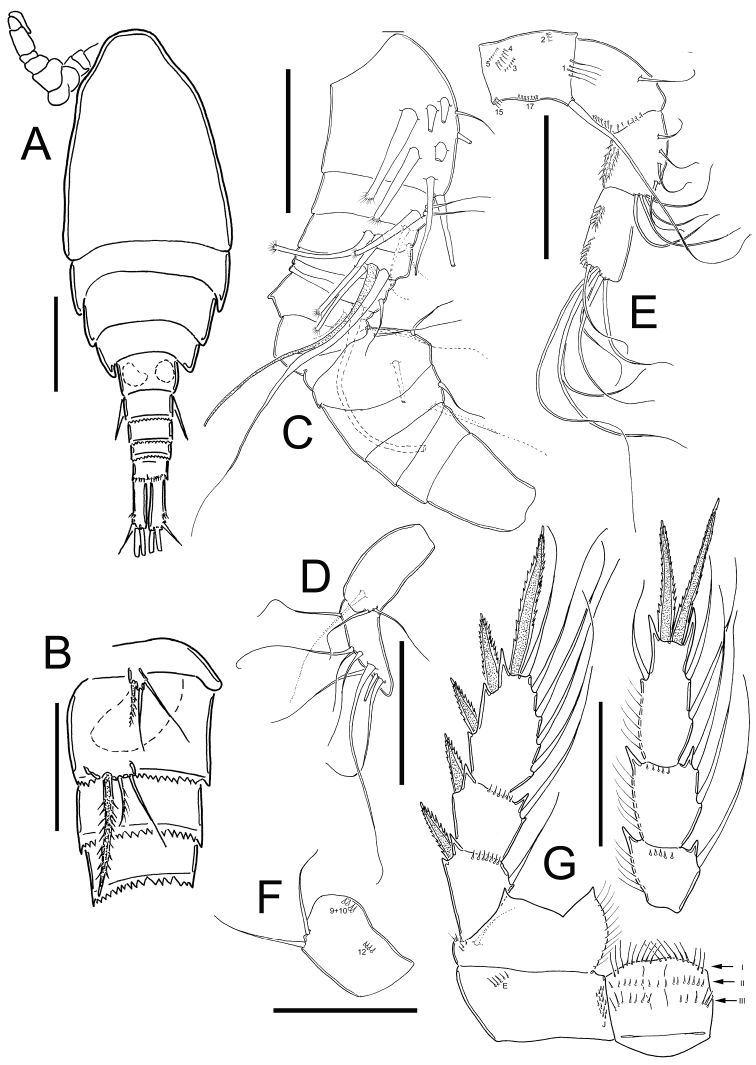
*Eucyclops tziscao* sp. n. **A–B** paratype **C–G** allotype from Laguna Tziscao, Chiapas. **A** Habitus, dorsal **B** P5, and P6 **C** Antennule, segments 1–14 **D** Antennule, segments 15–16 **E** Antenna, frontal **F** Antenna, caudal **G** P4, caudal. Scales bars: **B–G** = 50 µm; **A** = 100 µm.

*Male*: Habitus as in Fig. 4A; 509 µm of total body length excluding caudal setae. Body more slender than in female. Prosome symmetrical in dorsal view, representing 65% of total body length. Urosome short, representing 35% of total body length. Anal operculum slightly rounded and smooth. Caudal ramus 3.5 times longer than width; medial margin naked, strong spinules at insertion of lateral caudal seta (II) and outermost terminal caudal seta (III). Dorsal seta (VII) short 0.35 times the length of caudal ramus, and 0.75 times as long as outermost caudal seta (III). Ratio of innermost caudal seta (VI)/outermost caudal seta (III) is 1.6. Lateral caudal seta (II) inserted at 70% of caudal ramus. All the terminal caudal setae plumose.

*Antennule*: 16-segmented (Figs 4C, D), armament per segment as follows (s= seta, ms= modified seta, ae= aesthetasc, sp= spine): 1(7s+2ms); 2(3s+1ms); 3(1s+2ms); 4(1s+1ms+1ae); 5(0); 6(2s); 7(1s); 8(1s); 9(0); 10(3s); 11(2s); 12(0); 13(0); 14(0); 15(3s); 16(8s).

*Antenna* (Fig. 4E, F): Coxa (no seta), basis (2s + 1 seta representing Exp) plus 3-segmented Enp (first to third Enp with 1, 8, and 7 setae respectively). Basis ornamented with: N1 (4 hair-setae), N2 (4 small spinules), N3, N4, N5, N15, and N17 on frontal surface (Fig. 4E); and N9+10, and N12 on caudal surface (Fig. 4F).

*Legs 1–4*: Endopods and exopods of all swimming legs three-segmented (Table 2); P1-P3 as described in females.

*Leg 4* (Fig. 4G): Coxa, Bsp, and intercoxal sclerite as described in female, except for the distal row of spinules of intercoxal sclerite, which consists of 9 spinules longer and slender than in female (arrow of row I, in Fig. 4G). Enp3P4: 2.6 times as long as width; inner spine 1.2 times as long as outer spine, and 1.2 times as long as segment. No modified setae on fourth leg. Lateral seta of Enp3P4 inserted at 64.7% of segment length, lateral seta reaching the middle of outer spine.

*Leg 5* (Fig. 4B): One free segment subrectangular, 1.5 times longer than wide;

bearing one inner spine and two setae: outer seta subequal to median seta and 1.3 times longer than inner spine. Inner spine 1.8 times as long as segment.

*Leg 6* (Fig. 4B): Represented by small, low plate near lateral margin of genital somite with one strong and long inner spine and two unequal setae. Inner spine reaching the distal margin of fourth urosomite. Inner spine about 2.3 times longer than median seta and about 1.6 longer than outer seta.

#### Eucyclops angeli

Gutiérrez-Aguirre & Cervantes-Martínez sp. n.

http://zoobank.org/A2A11871-BE4A-48AC-9777-3735A0FF3EEA

http://species-id.net/wiki/Eucyclops_angeli

Figs 5 –9

##### Material examined.

Holotype: Adult *♀* specimen dissected, mounted in glycerin sealed with Entellan (ECO-CH-Z- 8967). Allotype: Adult *♂*, dissected, mounted in glycerin sealed with Entellan (ECO-CH-Z-8968). Paratypes: Eight adult *♀♀* undissected ethanol-preserved (90%) (ECO-CH-Z-8969); five adult *♀♀* and one adult *♂* undissected, ethanol preserved (90%) (MNHN-IU-2013-5970); four adult *♀♀*, undissected, ethanol preserved (90%) (CNCR 27841). Samples from type locality collected at 13. January. 2001 by A. Cervantes-Martínez, M. A. Gutiérrez-Aguirre and M. Elías-Gutiérrez.

##### Type locality.

Grassland near ECOSUR in San Cristóbal de las Casas City (Chiapas, México) (16°43'43"N, 92°38'14"W). At sampling the maximum depth was 1.48 m, the water temperature 21.5°C, and the dissolved oxygen 8.1 mg l^-1^.

##### Etymology.

This species is dedicated to Angel Cervantes Rivas, the first son of A C-M.

##### Description.

**Figure 5.**
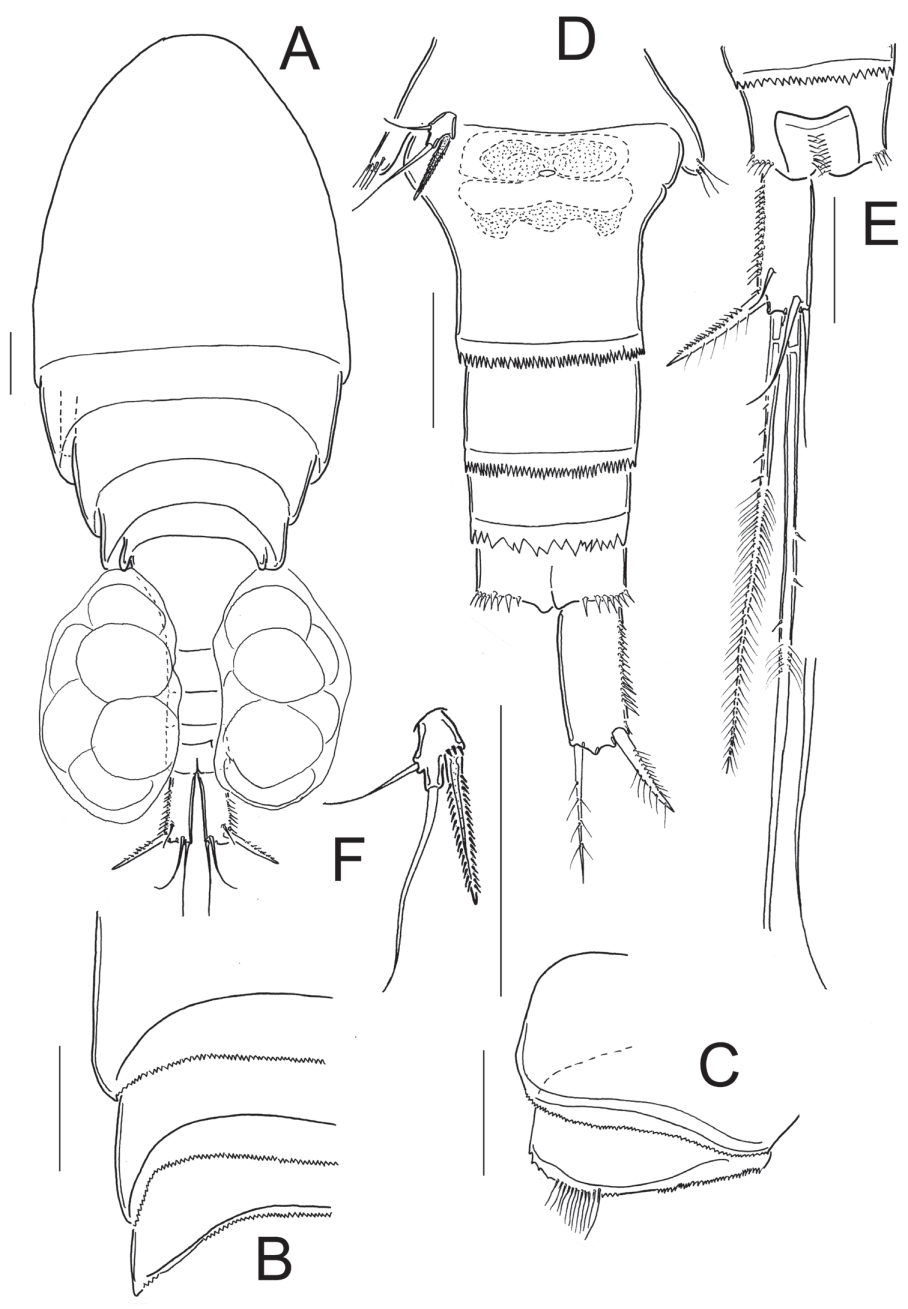
*Eucyclops angeli* sp. n. **A–C** paratype **D–F** holotype from grassland in San Cristóbal de las Casas, Chiapas. **A** Habitus, dorsal **B** Second to fourth prosomites, dorsal **C** Third and fourth prosomites, lateral **D** Urosome, ventral **E** Anal somite and one caudal ramus, dorsal **F** P5. Scale bars 50 µm.

*Female*: Habitus as in Fig. 5A; 600 µm of total body length excluding caudal setae. Prosome expanded at first and second somite, representing 58% of total body length, symmetrical in dorsal view (Figure 5A). Prosomal fringes serrated dorsally (Figure 5B); fourth prosomite with long, lateral, hair-setae (Fig. 5C). Five-segmented urosome, relatively elongated; first urosomite with long spinules on lateral margin; urosomal fringes strongly serrated (Fig. 5D). Posterior margin of anal somite with large spinules on ventral and dorsal surfaces, except for the medialmost section. Genital double-somite symmetrical, lateral arms of anterior part of seminal receptacle rounded; posterior part forming sinuous sac (Fig. 5D). Anal somite subequal in length to preanal somite and around 60% of caudal ramus length; with hair-setae in anal opening (Fig. 5D, E). Length/width ratio of caudal ramus 2.1; inner margin of caudal ramus naked, strong spines on lateral margin (serra) extending 62% of ramus length (Figure 5D). Dorsal seta (VII) relatively short, 0.83 times the length of caudal ramus, and 1.1 times as long as outermost terminal caudal seta (III). Innermost caudal seta (VI) 1.5 as long as outermost caudal seta (III). Lateral caudal seta (II) inserted at 71.6% of caudal ramus. Lateral seta (II) is 0.4–0.5 the length of outermost caudal seta (III). All terminal caudal setae plumose. Relative lengths of terminal caudal setae from outermost caudal seta to innermost caudal seta: 1.0: 3.9: 7.5: 1.2 (Fig. 5E).

**Figure 6.**
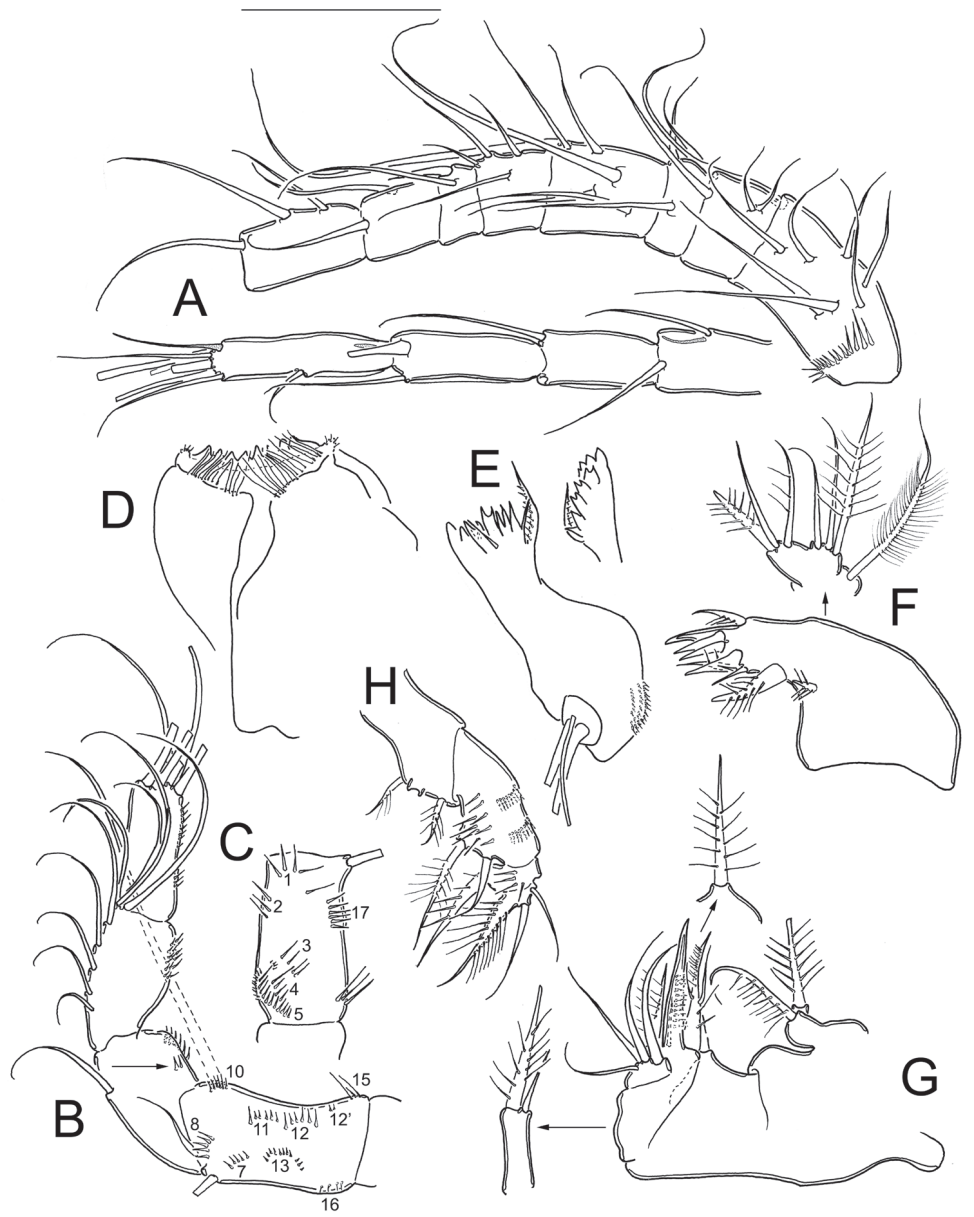
*Eucyclops angeli* sp. n. Holotype from grassland in San Cristóbal de las Casas, Chiapas. **A** Antennule **B** Antenna, caudal **C** Antenna, frontal **D** Labrum **E** Mandible **F** Maxillule, palp separated **G** Maxilla, proximal and distal endites of the coxa, separated **H** Maxilliped, frontal. Scale bar 50 µm.

*Antennule* (Fig. 6A): 12-segmented, tip reaching from middle to distal margin of second prosomite; smooth hyaline membrane on segments 10–12. The length ratio of segments 12/11 is 1.1. Armament per segment as follows (s= seta, ae= aesthetasc, sp= spine): 1(8s); 2(4s); 3(2s); 4(6s); 5(4s); 6(1s+1sp); 7(2s); 8(3s); 9(2s+1ae); 10(2s); 11(2s+1ae); 12(7s+1ae). Row of spinules on first segment: inner spinules shorter than outer spinules. Long spine on sixth segment, reaching the distal third of seventh antennular segment.

*Antenna* (Fig. 6B, C): Coxa (no seta), basis (2 s + 1 s representing Exp), plus 3-segmented Enp (first to third endopodite with 1, 9 and 7 setae respectively). Basis ornamented with: N1 (5 hair-setae), N2 (3 hair-setae), and N3, N4, N5, N17 (Fig. 6C) on frontal surface; and N7, N8, N10, N11, N12, N12’, N13, N15, and N16 on caudal surface (Fig. 6B). First to third endopodal segments with dense rows of spinules along lateral margin; Enp1 with additional row of 2 spinules on caudal surface (arrowed in Fig. 6B).

*Labrum* (Fig. 6D): Distal margin toothed. Ventral surface with long hair-setae. Two rounded lateral protuberances bearing spinules.

*Mandible* (Fig. 6E): With nine teeth on gnathobase, the innermost bi-toothed. Innermost margin with one spinulose seta. Palp with two long and one short setae. Three rows of tiny spinules next to palp.

*Maxillule* (Fig. 6F): Praecoxal arthrite with 3 chitinized claws and one spinulose seta on caudal side. Inner margin with two biserially plumose setae and four spiniform setae. Praecoxal surface naked. Palp naked, with Enp (3 long setae: one smooth, plus two plumose), Exp (3 long setae), and Bsp (with one plumose seta) (Fig. 6F).

*Maxilla* (Fig. 6G): Praecoxa and coxa partially fused. Praecoxal endite with two armed setae. Coxa naked and with two endites: proximal endite bearing one biserially plumose seta, distal endite with one long, plumose seta plus one short smooth seta. Claw-like basal endite with row of spinules on inner margin; one small seta inserted on caudal surface, and one chitinized armed seta inserted in front of claw-like endite. Endopod one-segmented bearing four smooth, long setae plus one plumose seta.

*Maxilliped* (Fig. 6H): Syncoxa with three setae bearing spiniform setules. Basis with two subequal setae and 9 spinules frontally. Two rows of acute spinules, each with 8 elements, arranged in semi-circular pattern on caudal surface. Endopod two-segmented: Enp1 with 4 basal spinules and one long seta fused to segment; Enp2 with three setae, longest seta biserially plumose and fused to Enp2.

**Figure 7.**
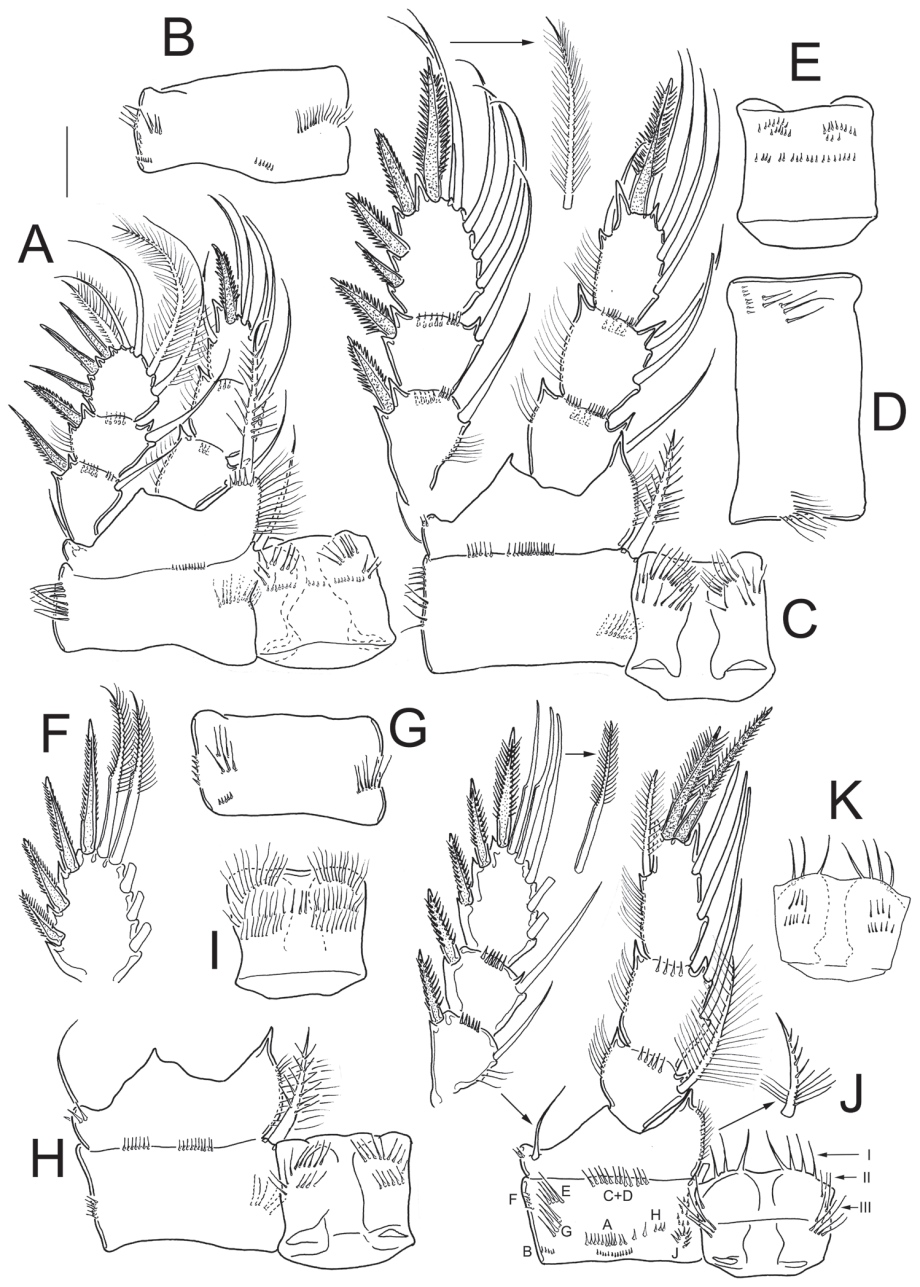
*Eucyclops angeli* sp. n. Holotype from grassland in San Cristóbal de las Casas, Chiapas. **A** P1, frontal **B** Coxa of P1, caudal **C** P2, frontal, Exp separated **D** Coxa of P2, caudal **E** Intercoxal sclerite of P2, caudal **F** Exp3P3 **G** Coxa of P3, caudal **H** Coxa, basis, and intercoxal sclerite of P3, frontal **I** Intercoxal sclerite P3, caudal **J** P4, caudal; exopod and coxal spine separated **K** Intercoxal sclerite of P4, frontal. Scale bar 50 µm.

*Legs 1–4*: With three-segmented Exps and Enps; intercoxal sclerites ornamented on frontal and caudal surfaces (Fig. 7). Armature formula of P1-P4 as in Table 3.

**Table 3.** *Eucyclops angeli* sp. n. Setation formula of the swimming legs in female, and male (spine in Roman numerals, seta in Arabic numerals).

	Coxa	Basis	Exp	Enp
P1	0-1	1-I	I-1; I-1; III-5	0-1; 0-2; 1-I-4
P2	0-I	1-0	I-1: I-1; IV-5	0-1; 0-2; 1-I-4
P3	0-I	1-0	I-1; I-1; IV-5	0-1; 0-2; 1-I-4
P4	0-I	1-0	I-1; I-1; III-5	0-1; 0-2; 1-II-2

*Leg 1* (Fig. 7A): Intercoxal sclerite armed with two arched rows of long spinules frontally and one row of tiny spinules caudally (Fig. 7A). Frontal surface of coxa with row of long spinules along lateral margin; long, feathered seta at mediodistal angle. Basis with one delicate outer seta, and one inner armed spine as long as Enp. Inner margin of Bsp hairy. Inner spine of Bsp with small basal spines, long setules proximally, and spine-like setules distally (Fig. 7A). Setae of Enp and Exp of P1 are unmodified, and regularly plumose in both edges (Fig. 7A). Caudal surface of coxa with four groups of spinules and one row of hair-like spinules near medial margin (Fig. 7B).

*Leg 2* (Figs 7C–E): Intercoxal sclerite with two rows of hair-setae on each of the two rounded projections on frontal surface (Fig. 7C), and three groups of spinules on caudal surface (Fig. 7E). Coxa with one row of long spinules along outer margin and two groups of spinules on distal margin on frontal surface (Fig. 7C). Lateral margin of coxa with two groups of long spinules, one group of short spinules, and one row of hair-setae in medial position on caudal surface (Fig. 7D). Armed coxopodal spine at mediodistal angle. Basis with one outer seta, inner margin hairy. All setae of Enp and Exp of P2 not constricted, plumose in both edges (Fig. 7C).

*Leg 3* (Fig. 7F–I): All setae on Exp and Enp as described in P2, except for the two distalmost setae of Exp3P3, which have very short setules along outer (constricted) edge (Fig. 7F). Caudal surface of P3 coxa with one row of tiny spinules along outer margin, one group of long spinules, one group of short spines, and one row of medial hair-setae (Fig. 7G). Caudal surface of intercoxal sclerite with three rows of hair- setae (Fig. 7I). On frontal surface, the ornamentation of coxa, Bsp, and intercoxal sclerite of P3, is similar to those in P2 (Fig. 7H).

*Leg 4* (Fig. 7J, K): Caudal surface of coxa with spinule ornamentation consisting of groups A–J. Coxal spine (inner seta) with heteronomous setulation: proximally with long setules, distally with spine-like setules; outer edge of coxal spine with a gap (Fig. 7J). Basis with delicate outer seta, and short hairs on inner margin. Three setae of Exp3P4 with constricted outer edge, and with short setules. Enp3P4 1.8 times as long as wide; inner spine 1.3 as long as outer spine and 1.3 as long as segment; outer spine 0.93 as long as segment. Caudal surface of intercoxal sclerite of P4 armed with 7 long denticles in position I, and long hair-setae in position II and III (Fig. 7J). Frontal surface of intercoxal sclerite of P4 with four rows of short spinules (Fig. 7K).

*Leg 5* (Fig. 5F): One free segment 1.4 times longer than wide; bearing one inner spine and two setae. Outer seta shorter than inner spine, relative lengths from outer seta to inner spine: 0.5: 1.6: 1 (Fig. 5F). Inner spine 2.0 times longer than segment.

**Figure 8.**
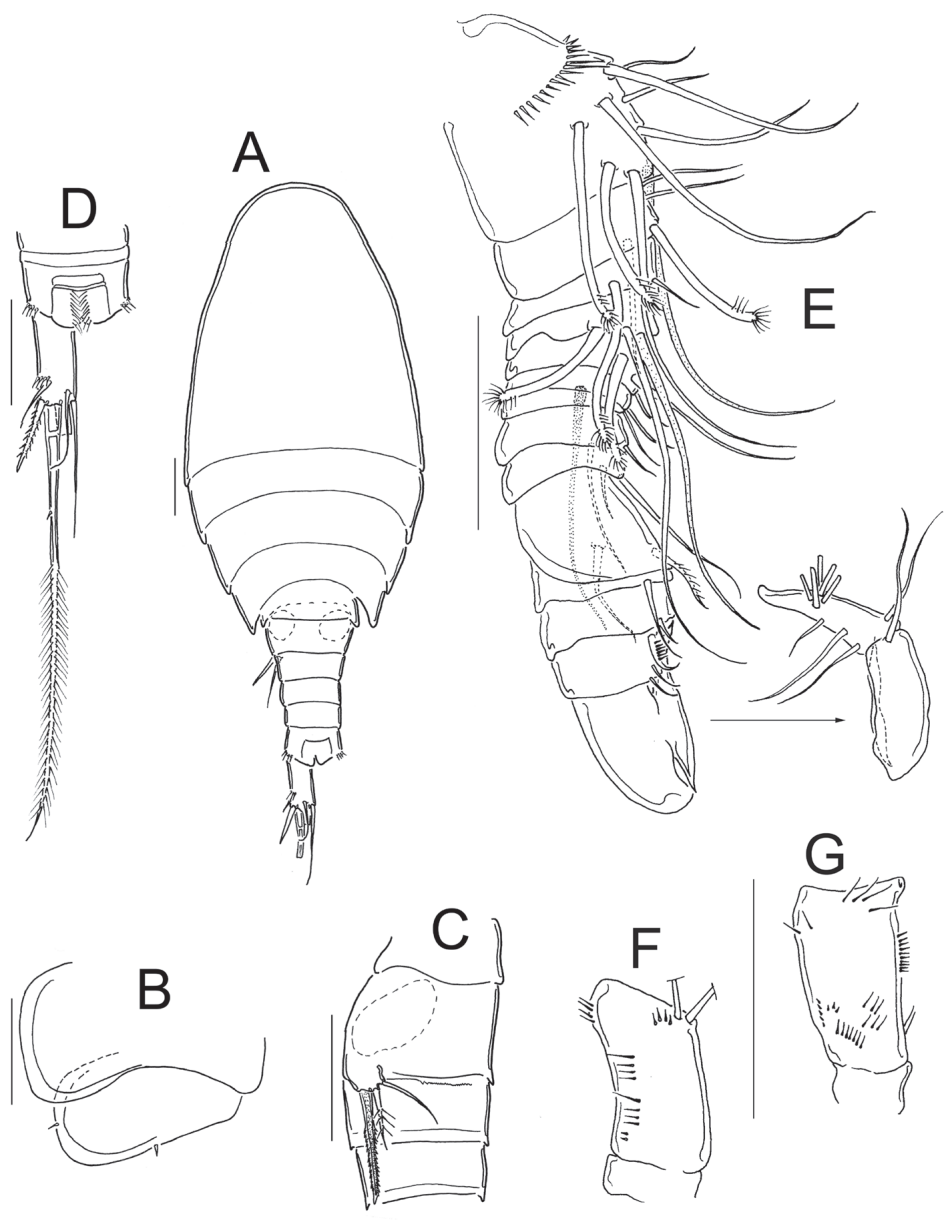
*Eucyclops angeli* sp. n. **A–B** paratype **C–F** allotype from grassland in San Cristóbal de las Casas, Chiapas. **A** Habitus, dorsal **B** Third, and fourth prosomites, lateral **C** First to fourth urosomites, lateral **D** Anal somite and caudal ramus, dorsal **E** Antennule, last two segments separated **F** Antenna, caudal **G** Antenna, frontal. Scale bars 50 µm.

*Male*: Habitus as in Fig. 8A; body length excluding caudal setae= 540–580 µm (n=4) average body length=552.9±15.56. Prosome symmetrical in dorsal view, representing 60–63% of total body length (Fig. 8A). Fourth prosomite with two spines, one spine on ventral margin, and one spine on posterior margin (Fig. 8B). Six-segmented urosome, relatively elongated; first urosomite naked on lateral margin (Fig. 8C); posterior margin of anal somite with a continuous (dorsally and ventrally) row of spinules (Fig. 8A, D). Anal region armed with two parallel rows of hair-setae; anal operculum slightly rounded and smooth (Fig. 8D). Caudal ramus 2.1±0.07 times longer than width (n=4); medial margin of caudal ramus naked, strong spines at insertion of lateral caudal seta (Fig. 8D). Innermost caudal seta (VI) 1.0–1.14 times longer than caudal ramus (n=3). Relative lengths of terminal caudal setae from outermost (III) to innermost (VI): 1.0: 5.7–6.4: 10.8–12.0: 1.45–1.6. Lateral caudal seta (II) 0.64–0.83 the length of outermost caudal seta (III) (Fig. 8D).

*Antennule* (Fig. 8E): 16-segmented, between segments 14–15 is the geniculation; armament per segment as follows (s= seta, modified seta= ms, ae= aesthetasc, sp= spine): 1(6s+2ms+1ae); 2(3s+1ms); 3(1s+1ms); 4(1s+1ms+1ae); 5(2s+1ms); 6(1s+1ae); 7(1s); 8(2s); 9(2s); 10(2s); 11(1s); 12(1s); 13(3s); 14(0); 15(1s); 16(9s). Row of spinules on first segment: inner spinules shorter than outer spines.

*Antenna*: As in female except for that the spinule groups N7, N13, and N16 are absent on caudal surface of antennal Bsp (Fig. 8F). Basis ornamented with: N1 (4 hair setae), N2 (2 hair setae) and spinules in groups N3, N4, N5, and N17 on frontal surface (Fig. 8G).

Labrum, mandible, maxillule, maxilla, and maxilliped as in female.

**Figure 9.**
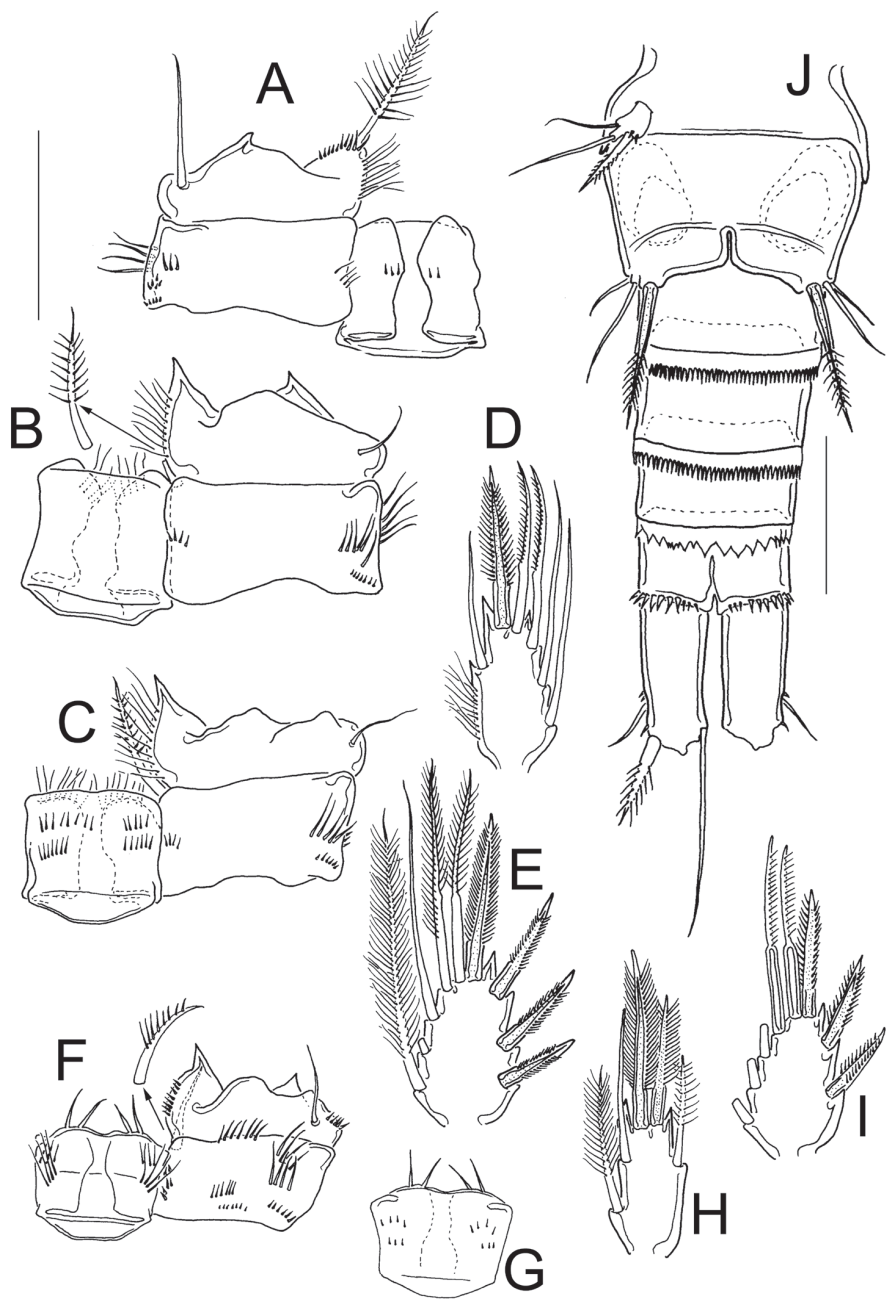
*Eucyclops angeli* sp. n. Allotype from grassland in San Cristóbal de las Casas, Chiapas. **A** Coxa, basis, and intercoxal sclerite of P1, caudal **B** Coxa, basis, and intercoxal sclerite of P2, caudal **C** Coxa, basis, and intercoxal sclerite of P3, caudal **D** Enp3P3 **E** Exp3P3 **F** Coxa, basis, and intercoxal sclerite of P4, caudal **G** Intercoxal sclerite of P4, frontal **H** Enp3P4 **I** Exp3P4 **J** Urosome, ventral. Scale bars 50 µm.

*Legs 1–4*: Exps and Enps three-segmented. Intercoxal sclerites armed as in Fig. 9A–C, F. Setation formula of swimming legs as in female (Table 3).

*Leg 1* (Fig. 9A): Intercoxal sclerite armed with two rows of tiny spinules on caudal surface. Ornamentation of Bsp, Enp, and Exp, as in female. Caudal surface of coxa with three groups of spinules and one row of hair-setae.

*Leg 2* (Fig. 9B): As in female, except for that intercoxal sclerite is naked on caudal surface, yet with two arched rows of long spinules on frontal surface.

*Leg 3* (Fig. 9C–E): Modified, intercoxal sclerite with rows of spinules caudally, and rows of hair-setae frontally (Fig. 9C). Two distalmost setae of Exp3P3 and Enp3P3 with constricted outer edges, and very short setules (Fig. 9D, E). Coxa and Bsp as described in female (Fig. 9C).

*Leg 4* (Fig. 9F–I): Coxa, basis, and intercoxal sclerite as in female; except for that entire outer margin of coxal seta is naked (Fig. 9F, G). Enp3P4: 2.07–2.25 (n=2) times as long as wide; inner apical spine 1.24–1.31 (n=2) as long as outer spine and 1.08–1.26 (n=2) times as long as segment (Fig. 9H). Two distal setae of Exp3P4 modified: chitinized, both edges constricted, and bearing short setules on outer edge (Fig. 9I).

*Leg* 5 (Fig. 9J): One free segment, 1.6 times longer than wide; and bearing three elements of which outer seta is slightly longer than that in female (subequal in length to inner spine) (Fig. 9J). Inner spine 1.8 times as long as segment.

*Leg 6* (Fig. 9J): Represented by a small, low plate near lateral margin of genital somite, armed with one inner spine, which is 1.7–1.87 times longer than outer seta, and 1.2–1.6 times longer than median seta. Inner spine of sixth leg reaching the distal margin of fourth urosomite.

#### Eucyclops festivus

Lindberg, 1955

http://species-id.net/wiki/Eucyclops_festivus

Figs 10 –11

Eucyclops festivus : Lindberg (1955), fig. 2a–d.
Eucyclops festivus : Suárez-Morales (2004), 617 p.
Eucyclops festivus : Mercado-Salas (2009), table 3, figs 138-139
Eucyclops pectinifer , Gutiérrez-Aguirre and Cervantes-Martínez (2013), table 1. Synonym.

##### Material examined.

One adult *♀* specimen dissected, mounted in glycerin sealed with Entellan. One adult *♂*, dissected, mounted in glycerin sealed with Entellan, and seven adult males undissected, ethanol preserved (90%) with a drop of glycerin, deposited in the senior author’s collection, at Universidad de Quintana Roo, Cozumel. Samples collected at 14. April. 2000 by A. Cervantes-Martínez, M. A. Gutiérrez-Aguirre and M. Elías-Gutiérrez in pond 3 to Laguna Montebello, Chiapas, México (16°06'42"N, 91°41'32"W). At sampling the maximum depth was 0.2 m; the water temperature 24°C and the dissolved oxygen 6.8 mg L^-1^.

##### Remarks.

**Figure 10.**
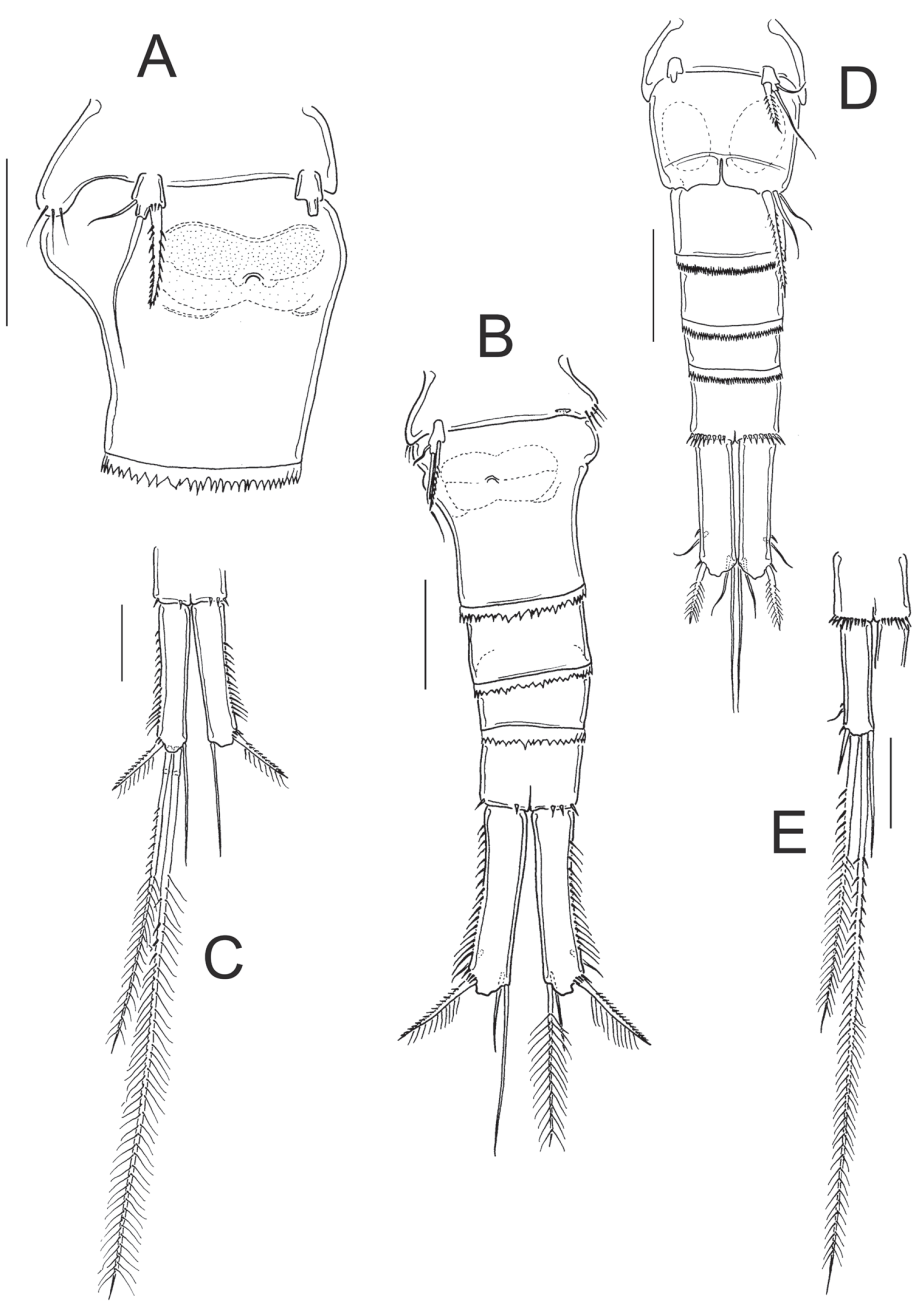
*Eucyclops festivus* Lindberg, 1955; from pond 3 to Laguna Montebello, Chiapas. **A** First urosomite, and genital double-somite, ventral **B** Urosome, ventral **C** Anal somite and caudal ramus, ventral **D** Urosome, ventral **E** Anal somite and caudal ramus, ventral. Scale bars 50 µm. **A–C** female; **D–E** male.

*Eucyclops festivus* has been recorded in North and Central Mexico (Suárez-Morales and Reid 1998, Mercado-Salas 2009). This is the southernmost record of the species in the country. Specimens from Chiapas were assigned to *Eucyclops festivus* because all the morphological characters, even the meristic features observed in the specimens from Chiapas are similar to those in the original description: in females and males the inner spine of fifth leg is 1.7–1.8 times longer than outermost seta, and the median seta is 1.5 times longer than the inner spine (Fig. 10A, B, D). The Fu length/width ratio is between 5–6 in the females, with spinules along the entire outer margin, and naked along inner margin (Fig. 10B, C). Caudal rami parallel in the male (Fig. 10D, E). The length ratio of innermost caudal seta (VI)/outermost terminal caudal seta (III) is 1.24±1.6 (Fig. 10C, E).

**Figure 11.**
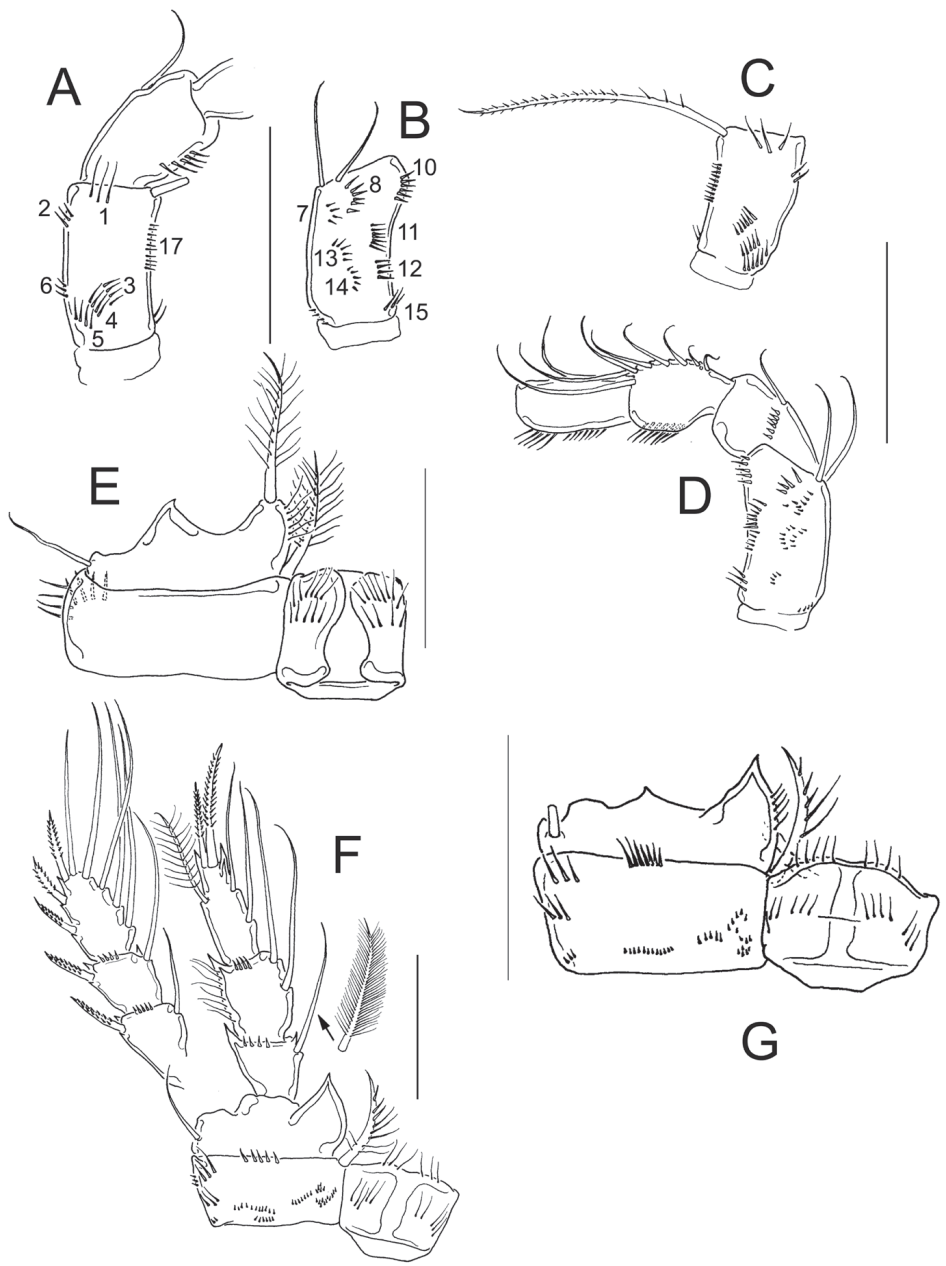
*Eucyclops festivus* Lindberg, 1955; from pond 3 to Laguna Montebello, Chiapas. **A** Antenna, frontal **B** Antenna, caudal **C** Antenna, frontal **D** Antenna, caudal **E** Coxa, basis, and intercoxal sclerite of P1, frontal **F** P4, caudal, Exp, and one inner seta separated **G** Coxa, basis, and intercoxal sclerite of P4, caudal. Scale bars 50 µm. **A, B, E, F** female; **C, D, G** male.

The antennal basis is adorned with the spinule groups N1, N2, N3, N4, N5, N6, and N17 on the frontal surface; whereas the groups N7, N8, N10, N11, N12, N13, N14, N15, and N16 are present on the caudal surface in female and male (Fig. 11A–D). Distal margin of the intercoxal sclerites in P1-P4 bear fine hair-setae (Fig. 11E–G). The length/width ratio of Enp3P4 is 2.2, the inner spine is 1.21 times longer than the segment, and the inner margin of BspP4 is naked in female (Fig. 11F).

Based on the presence of the group N6 on the frontal surface of antennal basis, the naked inner margin of BspP4, the long caudal rami, and the serrated hyaline membrane on the three distalmost segments of A1 in females, *Eucyclops festivus* is not included in the *serrulatus*-group.

## Discussion

The characters that allow us to include *Eucyclops tziscao* sp. n. and *Eucyclops angeli* sp. n. in the *serrulatus*-group sensu Alekseev and Defaye (2011) are as follows: 1) seminal receptacle bilobed, and the lobes subequal in size; 2) caudal ramus 2.0–7.0 times as long as wide, and with longitudinal row of spinules along most of the outer edge; 3) twelve-segmented antennule, with smooth hyaline membrane along distalmost segments; 4) frontal surface of antennal basis with 2–6 long spinules in N1, and variable number of spinules or strong denticles in the subdistal N2; 5) strong coxal spine of P4 with dense setules on inner side, and a large gap in setulation on outer side; and 6) one-segmented fifth leg with wide and strong, spine-like inner seta.

Following the identification key to the species of the *serrulatus*-group (Alekseev and Defaye 2011), *Eucyclops tziscao* was identified as *Eucyclops* cf. *bondi*, but after having performed a deeper analysis and compared our material to the types of *Eucyclops bondi* we concluded that our specimens belong to an another, though closely related species. The main characteristics that both species share are: a) on frontal surface of antennal basis, group N2 is represented by small spinules, b) the distal segment of P4 endopod with short inner distal seta, not reaching the end of outer apical spine and, c) caudal ramus with dorsal seta (seta VII) longer than outermost terminal caudal seta (III).

The differences between *Eucyclops tziscao* sp. n. and *Eucyclops bondi* are slight also in other characters, such asthe proportion of the caudal ramus (3.8–4.3 in *Eucyclops tziscao* sp. n., and 3.18–4.1 in *Eucyclops bondi*),the proportion of dorsal seta and caudal ramus length (0.63 in *Eucyclops tziscao* sp. n., and 0.73–0.80 in *Eucyclops bondi*) and the proportion of dorsal seta and innermost caudal seta length (0.8 in *Eucyclops tziscao* sp. n., and 1.0 in *Eucyclops bondi*).

Comparison of the type specimens of *Eucyclops bondi* to *Eucyclops tziscao* sp. n. revealed clear morphological separation of these taxa. One of the main distinguishing features between the two species is the length of the lateral seta on Enp3P4, which has already been reported as an important character in another species of the *serrulatus* group as in *Eucyclops delachauxi* (Kiefer, 1926). In *Eucyclops bondi* this seta exceeds the half length of the outer apical spine and it is not modified, while in *Eucyclops tziscao* sp. n. this seta is shorter, not reaching the half length of the outer apical spine, and it is modified as a strong, sclerotized blunt seta (arrowed in Fig. 3G). All setae on Enp3P4 are modified (strong, sclerotized and blunt) in *Eucyclops tziscao* sp. n., while in *Eucyclops bondi* they are not. In addition, the most apical seta of Exp3P4 is modified in *Eucyclops tziscao* while all setae are normal in *Eucyclops bondi*. All the setae on the swimming legs of *Eucyclops tziscao* sp. n. are shorter than in *Eucyclops bondi*. Finally, the length/width ratio of Enp3P4 is 2.6–3.0 in *Eucyclops tziscao* sp. n. while it is 2.4–2.6 in *Eucyclops bondi*.

The proportion of the inner apical spine and Enp3P4 segment length is slightly different in the species; it is 1.06 in the new species while itis 1.25 in *Eucyclops bondi*. Another useful character to differentiate taxa (as it has already been pointed out by Alekseev and Defaye 2011) is the setulation gap on the coxal spine of P4. Both the original drawings of Kiefer and the examination of the type material showed that in *Eucyclops bondi* the entire outer margin of the coxal seta is naked, while in *Eucyclops tziscao* sp. n. the apical region of the seta bears hair-like setules.

The use of male morphology in delineation of the species has been demonstrated in some genera of Eucyclopinae (e.g. *Paracyclops*, see Karaytug 1999; Karaytug and Boxshall 1999). One important feature that easily distinguishes *Eucyclops tziscao* sp. n. from *Eucyclops bondi* is the P6 armature, which is completely different in these two species. Our examinations showed that *Eucyclops bondi* has a unique sixth leg, in which the inner spine is relatively short in comparison to the median and outer setae: the proportion of the inner spine and outer seta length is 0.71 in *Eucyclops bondi*, while it is 1.5 in *Eucyclops tziscao* sp. n. also the proportion of inner spine and median seta lengthis about 1.07 in *Eucyclops bondi*, yet 2.5 in *Eucyclops tziscao* sp. n. In *Eucyclops bondi* the inner spine of sixth leg barely reaches the posterior margin of genital somite, while in *Eucyclops tziscao* sp. n. the spine extends up to the posterior margin of the fourth urosomite. All the records of *Eucyclops bondi* in the Americas should be re-evaluated considering this unique character (the very short inner spine of the male sixth leg) and also other morphological features, in order to clarify if they in fact belong to the species. For instance on the drawings of a material identified as *Eucyclops bondi* from Costa Rica (Collado et al. 1984) the sixth leg structure clearly does not correspond to the state present in the type of the species (cf. fig. 14 in Collado et al. 1984).

The species *Eucyclops pectinifer* seems to be closely related to *Eucyclops tziscao* sp. n., general body shape and some proportion of swimming legs are shared between both species. However the caudal ramus is shorter in *Eucyclops tziscao* sp. n. than in *Eucyclops pectinifer*, in the new species is 3.8-4.3 times longer than wide while in *Eucyclops pectinifer* is 4.5–5.0. Proportion of dorsal seta/length of caudal ramus is slightly longer in *Eucyclops tziscao* (0.6) than in *Eucyclops pectinifer* (0.4). Another difference between these two species is the shape of the anal operculum, in *Eucyclops pectinifer* is smooth and rounded, as in most *Eucyclops* species, while in *Eucyclops tziscao* sp. n. is rounded but strongly serrated (Fig. 2D). The ornamentation on frontal surface of antennal basis is similar in both species, they share groups N1 armed with long hairs, N2 bearing small and strong spinules, N3, N4, N5, N15 and N17; but the caudal surface of antennal basis is different: in *Eucyclops pectinifer* the groups of spinules N7, N13, N14 and an additional group of spinules below group N12 (see fig. 17-7 in Alekseev et al. 2006) are absent in *Eucyclops tziscao* sp. n.

Proportion of segments and elements in Enp3P4 are similar between species, length/width ratio of Enp3P4 is 3.2 in *Eucyclops pectinifer* while in *Eucyclops tziscao* sp. n. is 3.0; proportion of inner/outer apical spines of Enp3P4 is similar, 1.3 in *Eucyclops pectinifer* and 1.4 in *Eucyclops tziscao* sp. n.; in both species the lateral seta of Enp3P4 does not reach the half length of the outer apical spine, and it is modified as a strong, sclerotized blunt seta. But clear differences can be observed among the species in the fourth leg: in the intercoxal sclerite the row I in *Eucyclops pectinifer* bears fine, long hair-setae while in *Eucyclops tziscao* sp. n. it is armed with strong, short spinules. On the other hand, modified setae on swimming legs are present in both species but in *Eucyclops pectinifer* are present only in P4 while in *Eucyclops tziscao* sp. n. are present in P3 and P4.

In males, the antennular segments in *Eucyclops pectinifer* are 14 (Alekseev et al. 2006) while in *Eucyclops tziscao* 16. Proportional length of elements in P5 is clearly different among the species, in *Eucyclops tziscao* sp. n. outer and median setae are almost equal in size and clearly longer than inner spine; while in *Eucyclops pectinifer* median seta is more than two times longer than outer seta and inner spine, and the inner spine and outer seta are subequal in length. Sixth leg of males of both species differs slightly, in *Eucyclops tziscao* sp. n. the outer seta is two thirds the length of inner spine while in *Eucyclops pectinifer* this seta is shorter, being the half of size of the inner spine.

Another species that resembles *Eucyclops tziscao* sp. n. by sharing the modified setae on the third and fourth swimming legs and showing similar length and width proportion of the caudal ramus is *Eucyclops conrowae*. However when we compared the type material of *Eucyclops conrowae* deposited in Dr. Reid’s Collection (Smithsonian Institution) we found many differences. First of all *Eucyclops conrowae* is not a member of the *serrulatus*-group: the holotype and one paratype of *Eucyclops conrowae* do not have the groups N1 and N2 on the antennal basis (Table 4) whereas in *Eucyclops tizcao* sp. n. both groups are present in all the specimens here examined. Also, a group of spinules (J) is absent on the caudal surface of P4 coxa in *Eucyclops conrowae* (Table 5). Last, the seminal receptacle is completely different in these species;the posterior lobe is about twice the width of the anterior lobe in *Eucyclops conrowae*, whereas both lobes are approximately equal in size in the member taxa of the *serrulatus*-group.

**Table 4.** Comparison of the surface-ornamentation pattern of the antennal basis in some species of *Eucyclops*. Coding of the particular element follows Alekseev and Defaye (2011); Roman numerals, hairs; Arabic numerals, denticles; ?, structure not verified; NP, structure absent.

Species	1	2	3	4	5	6	7	8	9	10	11	12	12’	13	14	15	16	17
*Eucyclops tziscao* sp. n.	III-IV	5	5	6	8	NP	NP	4	5		5	5	NP	NP	NP	4	NP	10
*Eucyclops angeli* sp. n.	V	III	4	4	11	NP	5	5	NP	6	6	6	2	11	NP	3	4	6
*Eucyclops bondi* (type specimens)	?	?	?	?	?	?	?	?	?	?	?	?	?	?	?	?	?	?
*Eucyclops conrowae* (type specimens)	NP	NP	6	5	9	6	?	3	8		5	3	NP	NP	NP	3	NP	10
*Eucyclops serrulatus* (Alekseev and Defaye 2011)	IV-IX	I-IV	6–10	7–9	12–18	NP	3–5	5–8	NP	NP	5–6	6–8	NP	0–4	3–8	4–7	0	10–13
*Eucyclops albuferensis* (Alekseev 2008)	VII	III	8	3	3	NP	8	5	NP	7	9	9	NP	7	10	6	7	18
*Eucyclops dumonti* (Alekseev 2000)	NP	NP	NP	7	15	NP	NP	5	NP	4	8	8–9	NP	NP	NP	3	4	10–12

**Table 5.** Comparison of the surface-ornamentation pattern of the P4 coxa in some species of *Eucyclops*. Coding of the particular elementsfollows Alekseev and Defaye (2011) (see fig. 2C in Alekseev and Defaye 2011). Y, present; N, absent; H, hairs; LH, long hairs; SH, short hairs; D, denticles.

Species	A	B	C+D	E	F	G	H	J	I	II	III	Gap on coxal spine	Hair-like setae on basipodite
*Eucyclops tziscao* sp. n.	Y	N	11	5–6	Y	N	Y	Y	D	D	D	Y	Y
*Eucyclops angeli* sp. n.	Y	6	10–12	4	Y	Y	Y	Y	LD	LH	LH	Y	Y
*Eucyclops bondi* (type specimens)	Y	5	12	4	Y	Y	Y	Y	D	SH	LH	Y	Y
*Eucyclops conrowae* (type specimens)	Y	N	10	6	N	Y	Y	N	D	D	D	Y	Y
*Eucyclops serrulatus* (Alekseev and Defaye 2011)	Y	4–5	12–14	2–4	N	N	Y	Y	LH, SH	SH	LH	Y	Y
*Eucyclops albuferensis* (Alekseev 2008)	Y	N	13	5	Y	Y	Y	Y	SH	SH	SH	Y	Y
*Eucyclops dumonti* (Alekseev 2000)	Y	2–3	10–12	2	Y	Y	Y	Y	SH	SH	SH	N	N

*Eucyclops angeli* sp. n. can be distinguished from all the other *Eucyclops* species by: the short caudal rami; long spine on the sixth antennular segment; presence of an additional group of spinules (N12’) on the caudal surface of antennal basis; presence of long hair-setae in females, and short spinules in males on the lateral margin of fourth prosomite; rich surface ornamentation of P1-P4 intercoxal sclerites both on the frontal and caudal surfaces, long denticles in group I on the intercoxal sclerite of P4; modified distal setae of Exp3P3 and Exp3P4 in females and males; as well as the short outer seta of P5.

In the Americas there are some other species which have similarly short caudal rami. *Eucyclops breviramatus* Loeffler, 1963 in Ecuador, l/w: 2.3–2.6 (Loeffler 1963); and *Eucyclops siolii* Herbst, 1962 in Brazil and Venezuela, l/w: 2.18 (Herbst 1962). The morphological characters of A1, A2, P4, P5, and the caudal ramus indicate that *Eucyclops angeli* sp. n. belongs to the *serrulatus*-group as defined by Alekseev et al. (2006) and Alekseev and Defaye (2011). Relying upon the information given in Loeffler’s description (1963) on the morphology of A1 (12-segmented), P5 (medial spine longer than free segment), and caudal rami (with longitudinal row of spinules along most of outer edge), *Eucyclops breviramatus* can be considered as member of the *serrulatus*-group too. Even though the microcharacters of the antennule, antenna, intercoxal sclerites, and P1-P4 coxa are unknown in *Eucyclops breviramatus* (see Loeffler 1963), there are other characteristics that can differentiate *Eucyclops breviramatus* from *Eucyclops angeli* sp. n. for instance, the length/width ratio of Enp3P4 (1.81–1.97 in *Eucyclops angeli* vs. 1.4–1.5 in *Eucyclops breviramatus*); the short outer seta of P5 in *Eucyclops angeli* (outer seta subequal or slightly longer than inner spine in *Eucyclops breviramatus*); and the different length proportion of the caudal setae: whereas the relative length of the terminal caudal setae from outermost to innermost is 1.0: 2.98–3.55: 5.4–6.5: 1.06–1.16 in *Eucyclops breviramatus*, clearly the inner median and inner outer setae are longer in *Eucyclops angeli* sp. n. because the relative length is: 1.0: 3.9–4.5: 7.5–9.6: 1.2–1.4.

Another American species with very short caudal rami is *Eucyclops siolii*, yet likely this species does not belong to the *serrulatus*-group. *Eucyclops siolii* has a very short inner spine on the leg 5 (proportion of the spine and free segment length, 0.75), the coxal spine of P4 bears hair-setules on the inner and outer margin, and the intercoxal sclerite of P4 is only adorned with the spinule groups I and II on the caudal surface (see Herbst 1962).

*Eucyclops conrowae* shares the modified distal setae on the exo- and endopod of P3 and P4 with *Eucyclops angeli*, yet analysis of the holotype and paratype of the former species revealed several differences on the antennal basis, the coxa, and intercoxal sclerite of P4, which separate these two taxa. In *Eucyclops conrowae* the groups N1, N2, N13, N14, and N16 are absent on the antennal basis (Table 4), the spiny group J is absent on the P4 coxa, and the P4 intercoxal sclerite bears only denticles (Table 5).

Recently Alekseev (2008) described *Eucyclops albuferensis*, from Valencia Spain, which is similar to *Eucyclops angeli* sp. n. in the ornamentation of the caudal surface of P4 coxa, setulation of the P4 coxal spine, and ornamentation of the antennal basis. However unlike *Eucyclops angeli*, *Eucyclops albuferensis* only has short hairs in the groups I, and II on P4 intercoxal sclerite, whereas *Eucyclops angeli* has long denticles in group I, and long hairs in groups II, and III. Also, *Eucyclops albuferensis* has longer caudal rami in the female (about 5 times as long as wide), and it does not have modified setae-spines on Enp3P3 and Enp3P4. Last, in the male of *Eucyclops albuferensis* the inner spine of the sixth leg, does not reach the distal margin of the fourth urosomite, as it does in *Eucyclops angeli* sp. n.

*Eucyclops dumonti* Alekseev, 2000 is another species with short caudal rami (about 2.9 times as long as wide) (Alekseev 2000) which lives in a spring lake in Central Mongolia. *Eucyclops dumonti* differs from *Eucyclops angeli* sp. n. in the intercoxal sclerites of P1-P4, which have much less surface structures in *Eucyclops dumonti*: P1-P4 intercoxal sclerites bears more groups of long hairs in *Eucyclops angeli*. Moreover, the inner margin of P4 basis is naked in the female of *Eucyclops dumonti*, yet it has short hair-like spinules in *Eucyclops angeli*. The spinule groups N7 and N13 (caudal surface), and N1 and N2 (frontal surface) that are present on the antennal basis in *Eucyclops angeli* sp. n., are absent in *Eucyclops dumonti*. Finally, in the male of *Eucyclops dumonti* the tip of the medial spine of P6 reaches the distal margin of the third urosomite, yet in *Eucyclops angeli* this spine is longer, reaching the fourth urosomite.

*Eucyclops echinatus* (Kiefer 1926) distributed in Africa (Angola, Democratic Republic of Congo, Ivory Coast, Kenya, and Madagascar) is another species with short caudal rami (length/width, 2.22–2.26), but it differs from *Eucyclops angeli* sp. n. in the ornamentation of the dorsal and medial surface of the caudal rami (with short denticles in *Eucyclops echinatus*) (Kiefer 1926), and the relative length of the outer seta of P5: in *Eucyclops echinatus* the outer seta is 1.3 times longer than the inner spine, whereas in *Eucyclops angeli* sp. n., this seta is 0.6–0.9 times the length of the inner spine.

*Eucyclops festivus* has so far been known from North and Central regions of Mexico, mainly from the littoral region of dams, and permanent or ephemeral ponds (Lindberg 1955, Mercado-Salas 2009). The southernmost confirmed record in Mexico is that from Hidalgo State (Lindberg 1955, Suárez-Morales and Reid 1998), thus the known distribution of this species in Mexico, including our present finding in Chiapas, extends from 21°N to 16°N.

## Conclusion

The two new species here described belong to the *Eucyclops serrulatus*-group. Due to the complex taxonomy and uncertain status of most species in *Eucyclops*, a morphological revision should be performed in the Americas. The genus has been revised in some regions of the world, but many American records still need verification. The use of new morphological characters facilitated better delineation of *Eucyclops* species which in turn resulted in better knowledge of the zoogeography of the genus – contrarily to what was believed, most species have well defined restricted geographic distribution, and they are not cosmopolitan.

With the description of the two new species, the number of *Eucyclops* taxa in Mexico now reaches 15 species, however several records referring to the problematic taxa should be revised. We strongly recommend to use males in the identification of *Eucyclops* species, because they present highly informative characters. Male morphology could help to clarify the identity of some problematic species, as shown for *Eucyclops bondi*.

## Supplementary Material

XML Treatment for Eucyclops tziscao

XML Treatment for Eucyclops angeli

XML Treatment for Eucyclops festivus
